# Electricity and disinfectant production from wastewater: Microbial Fuel Cell as a self-powered electrolyser

**DOI:** 10.1038/srep25571

**Published:** 2016-05-12

**Authors:** Iwona Gajda, John Greenman, Chris Melhuish, Ioannis A. Ieropoulos

**Affiliations:** 1 Bristol BioEnergy Centre, Bristol Robotics Laboratory, University of the West of England, BS16 1QY, UK; 2 Biological, Biomedical and Analytical Sciences, University of the West of England, BS16 1QY, UK

## Abstract

This study presents a simple and sustainable Microbial Fuel Cell as a standalone, self-powered reactor for *in situ* wastewater electrolysis, recovering nitrogen from wastewater. A process is proposed whereby the MFC electrical performance drives the electrolysis of wastewater towards the self-generation of catholyte within the same reactor. The MFCs were designed to harvest the generated catholyte in the internal chamber, which showed that liquid production rates are largely proportional to electrical current generation. The catholyte demonstrated bactericidal properties, compared to the control (open-circuit) diffusate, and reduced observable biofilm formation on the cathode electrode. Killing effects were confirmed using bacterial kill curves constructed by exposing a bioluminescent *Escherichia coli* target, as a surrogate coliform, to catholyte where a rapid kill rate was observed. Therefore, MFCs could serve as a water recovery system, a disinfectant/cleaner generator that limits undesired biofilm formation and as a washing agent in waterless urinals to improve sanitation. This simple and ready to implement MFC system can convert organic waste directly into electricity and self-driven nitrogen along with water recovery. This could lead to the development of energy positive bioprocesses for sustainable wastewater treatment.

Biomass and household waste have been identified as resources that demonstrate great promise for the UK bioenergy sector, in terms of their availability, quantity and bioenergy potential^1^. Generally a sustainable wastewater management system could be based on policy including reuse, recovery and recycling rather than developing new water supply sources, especially when used in regions where freshwater is scarce^2^. Water and nutrient recovery can be achieved through electrochemical processes such as desalination, however despite recent improvements^3^, it is still relatively expensive and subject to membrane biofouling. The last two decades have seen considerable improvements in the development of the electrochemical processes^4^ for wastewater treatment, remediation and disinfection^5^. Wastewater electrochemical cells (WEC) have been proposed for waste treatment to improve sanitation in remote locations lacking conventional urban infrastructure^6^. These WEC systems require a supply of electricity in order to electrolytically sanitise wastewater^7^, including urine^6^. To further improve sanitation, electrochemical chlor-alkali systems have been developed as a quick and efficient method of producing electrochemically activated solutions (ECAS) in the form of acidic anolyte and alkaline catholyte. Chlor-alkali cells produce chlorine and caustic soda through electrolysis of a salt solution^8^ where hydrated Na + ions migrate through the ion exchange membrane from the anolyte into the catholyte to react with the hydroxyl ions. The anode and cathode in ECAS systems are usually separated by a ceramic diaphragm^9^ producing electrolysed solutions, that can have potent bactericidal properties (acid) and strong reducing potential (basic)^10^,^11^. An electrolysed basic solution (pH > 11) is produced from the cathode side, which may be used as a cleaning agent^12^ as it demonstrates excellent particle removal abilities^13^ and reduction of microbial populations^14^.

The integration of microbiology and electrochemistry has been demonstrated in the field of Microbial Electrolysis Cells (MECs) where an external supply of electricity drives the production of chemicals such as caustic solutions^15^,^16^,^17^,^18^, biosynthesis and nutrient recovery^19^.

However, a more sustainable application of electrochemical systems is presented by the microbial fuel cell (MFC) as a direct electricity producer suitable for wastewater treatment^20^,^21^,^22^. The technology has been developed for the recovery of water^23^, nutrients^24^ and is suitable for practical applications^25^,^26^,^27^. MFCs involve the direct generation of electrons from organic matter, due to the bio-catalytic activity of microorganisms in the anode chamber, and the reduction of oxygen in a cathode chamber, by the electrons (via a circuit) and protons (via a semipermeable membrane or separator) coming from the anode, to complete the circuit. As a sustainable bioelectrochemical system, MFCs have the potential to transform waste remediation with net positive electrical energy gain.

However, it has been recently proposed that MFC driven electrosynthesis has the ability to generate electricity as well as simultaneously produce valuable chemicals such as caustic catholyte solution^28^. This is due to the oxygen reduction reaction (ORR) and electroosmotically produced catholyte, directly on the cathode surface^28^,^29^,^30^. Electroosmosis represents the motion of liquid through a porous material under the influence of electric field and during MFC operation, as the protons and cations migrate through the membrane to the cathode, water is simultaneously moved by the electroosmotic drag. The cogeneration of electricity and caustic solutions is a very attractive and sustainable option for wastewater treatment and requires further investigation. It opens up numerous opportunities including catholyte reuse, as an active basic solution, presenting the MFC system as an *in situ* wastewater electrolyser with a net production of electricity. It is a promising, low cost solution for disinfection applications, such as MFC-powered urinals^31^, particularly in the Developing World to improve sanitation and help control microbial pathogens. This work is aiming to explore the principal properties of the Microbial Fuel Cell as an innovative self-powered ECAS machine for the generation of electrolysed basic solution from wastewater. The use of generated *in situ* biocidal catholyte produced from wastewater has never been presented before. This is to show that the microbial bio-engine in the anode is driving electrolysis, and at the same time generating – rather than consuming – electrical current. This is distinctly different to any power-consuming electrolysis systems presented thus far.

## Results and Discussion

### Performance and catholyte extraction

**Figure 1:**
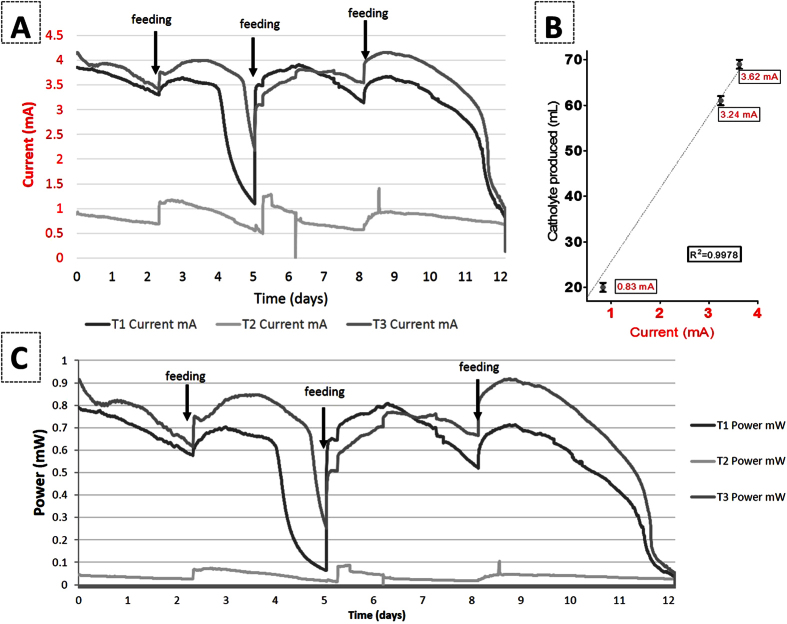
Current output over a 12- day period during which the catholyte accumulated in the cathode, the arrows indicate the addition of fresh substrate to the anode (**A**). Catholyte generated plotted against current showing linear correlation (**B**). Power output over a 12-day trial (**C**).

The experiments started with fully inoculated and matured anodes with (naturally) empty cathode chambers. As shown in Fig. 1A-1C, the performance was monitored over a 12-day period after which the catholyte was collected from the inner chamber. During this time, the substrate was replenished by adding feedstock (sludge + ammonium acetate) to the anode chambers, due to the loss of anolyte and decrease in performance. At the end of the test the average current generation was 3.24 mA, 0.83 mA and 3.62 mA for T1, T2 and T3, respectively. T2 was clearly underperforming and although the cause of this behaviour was not determined (see polarisation and power curves in Supplementary Information), it is possible to assume that this was due to the poor physical contact of the cathode electrode with the inner terracotta wall. However, the aim of this line of work was to study the principle of the catholyte extraction so the MFC, albeit underperforming, was still included. The catholyte volumes collected were 73, 32 and 81 mL for T1, T2 and T3, respectively showing a linear correlation with current (Fig. 1B). For the control open circuit MFCs, catholyte volumes of 12, 11 and 13 mL were collected for T4, T5 and T6, respectively. The accumulation of a small volume of catholyte under open circuit conditions is due to the passive osmotic pressure as shown previously^29^,^30^ and therefore the data in Fig. 1B have been normalised accordingly, discounting the volume generated under passive diffusion. This suggests that the rate of catholyte generation is a function of MFC performance relating to the rate of charge transfer in the system. Moreover the accumulation of liquid in the cathode does not hinder the MFC performance; in contrast it might actually improve it as previously shown^32^. From previous work and also from the current study, we could assume that the electroosmosis is indeed the dominant process, especially when this has been studied in detail in evaporation controlled conditions^30^. The role of water synthesis vs electroosmosis is a very interesting aspect and it will form part of a future study; initial calculation of the Coulombic yield of synthesised water through ORR suggested only 0.4 g of H_2_O produced over 14 days of MFC operation (data not published) indicating that the synthesised catholyte is not significant.

**Figure 2:**
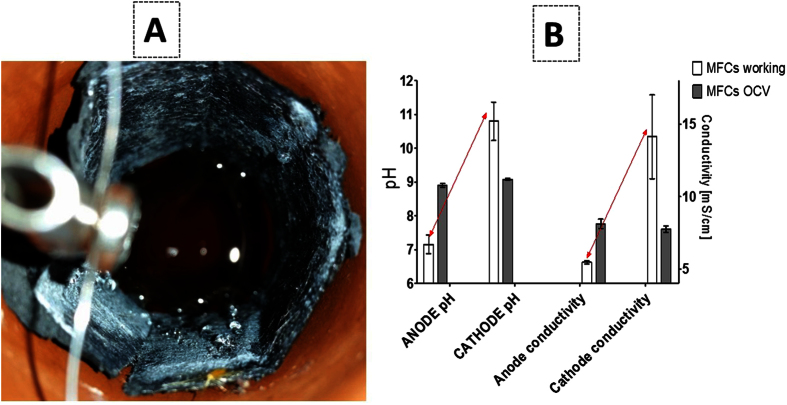
Photograph showing droplets formed inside the MFC cylinder accumulating liquid catholyte (**A**). Bar chart showing pH and conductivity measurements of the anolyte and catholyte, when the MFCs were producing power vs. the control MFC in open circuit conditions (**B**) data shown are the average (with error bars) from the three working MFCs, T1, T2 and T3 and three control MFCs T4, T5 and T6.

The quality of the catholyte was investigated and it was shown to be a transparent liquid formed as droplets on the surface of the cathode electrode (Fig. 2A) with a pH in the range of 11.91, 10.07 and 10.42 and conductivity of 11.99 mS/cm, 19.87 mS/cm, 10.54 mS/cm for T1, T2 and T3, respectively (Fig. 2B), while the initial anolyte pH and conductivity values were 7.16 and 6.67 mS/cm, respectively. This indicated that the MFC operation increased both the pH and conductivity of the actively transported catholyte, in comparison with the control MFCs that were left open circuit.

The catholyte of the working MFCs had a very strong odour of ammonia. It is suspected that this is due to the active migration of ammonium cations from the anode to the cathode^33^. Strong ammonia odour suggests that NH_4_^+^ has been stripped from the anode solution and converted to NH_3_ gas in the cathode, due to the high pH in the cathode chamber (Fig. 2B)^34^. In this set-up the ammonium ions were concentrated in the inner cathode chamber, which suggests a high concentration of ammonia in the catholyte (ammonium hydroxide). As can be seen from the pH/conductivity values in Fig. 2B, the catholyte properties of the OCV MFCs, were similar to those of the anolyte. This is suggesting that a small amount of liquid diffuses passively from the anode to the cathode, without any ‘active filtration’ from the ceramic separator. For the MFCs under the 53 Ω load (working MFCs), the amount of catholyte generated is up to 7 times higher than that from the OCV MFCs, and the properties of the catholyte are significantly different to the anolyte. In previous reports where sodium acetate was used as a substrate, pH in the cathode was elevated to >12^28^. Here with ammonium acetate used as the fuel, ammonium is the cation that is being actively transported from the anode to the cathode. With the ORR generating OH–35 and increasing the pH in the cathode, it is suspected that ammonium hydroxide (NH_4_OH) is formed. The latter is a non-metallic weak base, which does not reach high pH levels in comparison to sodium hydroxide, as previously reported^29^.

### Combined nitrogen and carbon removal with energy generation

**Figure 3:**
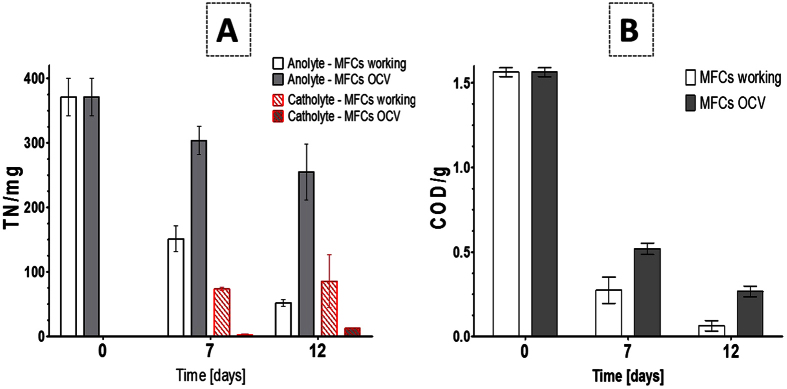
Total Nitrogen (TN) removal and recovery in the cathode from the working and control MFCs (**A**). COD reduction in working and OCV MFCs during the 12 day period (**B**).

The double chamber bioreactor with the inner cathode was employed to accomplish simultaneous electricity generation and catholyte formation and the experimental results have demonstrated a successful MFC operation. When the feed solution (sludge mixed with 0.1 M ammonium acetate) was supplied to the MFCs in the anode, the MFCs were monitored for COD and Total Nitrogen (TN) removal in batch mode over the 12-day period. A maximum of 77% TN removal was recorded from the anolyte of the working MFCs (Fig. 3A) and transported into the cathodic chamber, compared with the OCV MFCs, which show a TN reduction of 31%. Moreover, the actively transported ammonium with liquid water can be harvested in the cathodic chamber showing 18% of nitrogen recovery (working MFCs) in comparison with 2% (MFCs under OCV) therefore the data suggest that nitrogen in the catholyte is not yet fully recovered. Figure 3B shows a 95% COD reduction from the working MFCs. Also it may be noted that the high COD removal in the open circuit control reactors was due to natural, biological oxidation occurring during the 12 day trial. The biological oxidation at the cathode was not studied and since the catholyte properties are similar to the anolyte in OCV conditions, it is fair to assume that the anolyte (acetate based) was passively diffusing to the cathode where it would be further oxidised. The MFC reactors are not designed to be air-tight, and there is bound to be room for improvement, however there was no performance deterioration because of this, which implies that the system works as a column.

Since ammonia may exist in the solution either in non-ionized form (NH_3_) and/or as the ionized form (NH_4_^+^), an efficient stripping requires a pH adjustment above 10. The relative proportion of the two forms in aqueous solutions is mainly affected by pH and temperature, with a dissociation constant (pKa) of NH_4_^+^/NH_3_ equal to 9.25 at 25 °C^36^. Ammonia stripping is a simple process that lowers the ammonia content in wastewater by adding alkaline compounds^37^. Ammonium ions exist in equilibrium with ammonia and below pH 7 are fully protonated and highly soluble, thus remaining in the liquid phase. However, above pH 7 the percentage of the non-protonated form (dissolved gas) will increase with increasing pH (caused by MFC cathode) and ammonium is transferred from the waste stream into the air. In the traditional ammonia stripping process, caustic soda is added to wastewater until the pH reaches between 10.8 to 11.5 standard units, which convert ammonium hydroxide ions to ammonia gas. Here, the catholyte pH increase, as well as the detected strong ammonia odour, suggest that the catholyte is actively removing ammonia from wastewater.

Ammonium recovery has been studied in MFCs^34^ however the process has to be further optimised to practically implement the technology^38^. Ammonium was found to diffuse through the membrane, causing an elevated level of ammonium in the final effluent thus showing an inefficient removal^39^. Recovering nitrogen from different types of waste through liquid extraction is considered to be a more sustainable approach than removing it at the expense of natural resources and significant costs of nitrogen fixation. The recovered nitrogen may be applied as a fertilizer to agricultural production, with the appropriate selectivity and concentration of elemental cations, with the added bonus of limited sludge volume production.

### Bacterial-limiting properties of extracted catholyte

**Figure 4:**
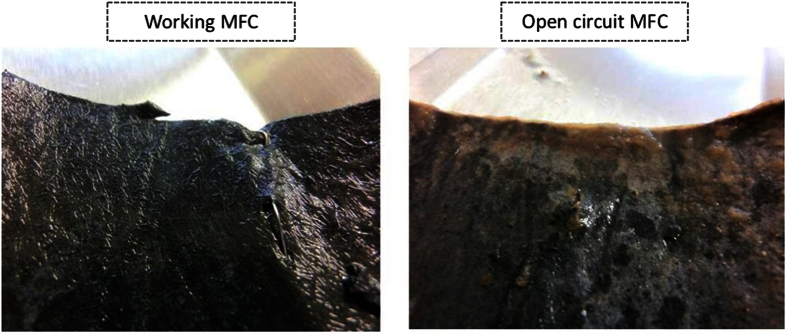
Gas diffusion side of the cathode electrode of the loaded (working) MFCs (left) and open circuit MFCs (right). Biofilm growth was observed only on the OCV MFCs i.e. that do not produce electricity.

Cathode electrode observation showed that in all MFCs that were generating electricity (i.e. working MFCs) the electrodes were visually clear of any biofilm. The open circuit MFCs on the other hand, showed a markedly different response with thick biofilm growth, over the same period, as shown in Fig. 4. This suggested that the actively produced caustic catholyte was inhibiting any growth, and may serve as a disinfectant or cleaner to prevent biofouling of the cathode electrode as well as the membrane.

**Figure 5:**
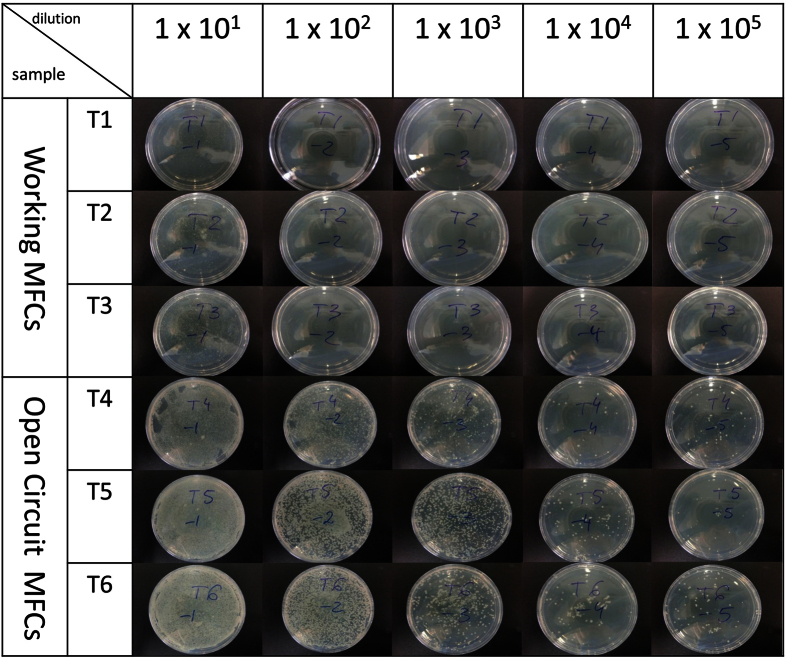
Catholyte samples from working (under load) and open circuit MFCs in serial dilutions cultivated on nutrient agar plates and using the standard sub-culture method.

**Figure 6:**
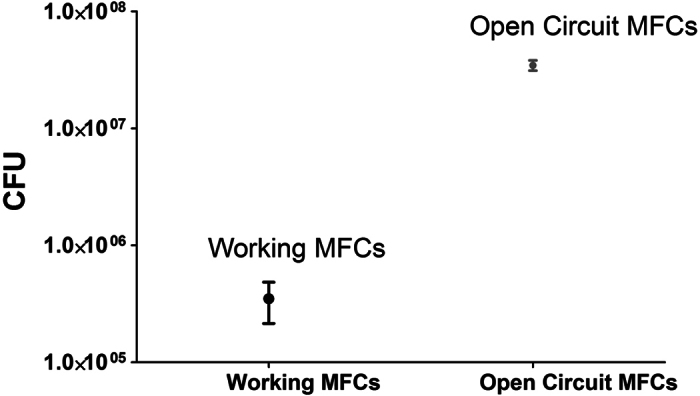
CFU count obtained from the catholyte samples in working and control conditions.

Total Viable Counts confirmed that the catholyte obtained from the working MFCs showed anti-growth properties probably due to increased pH and conductivity of the generated active basic solution. The comparative analysis in Fig. 5 shows visible microbial growth prevention in the catholyte from a working MFC, with an almost 4-log reduction of microbial growth, shown in Fig. 6, in comparison to the OCV control. The density of microbial cells suggests that these are alkalophilic species with low diversity and yield at 5.5 × 10^5^ cfu/mL in contrast to those obtained from the open circuit MFC at 1.5 × 10^9^ cfu/mL (Fig. 6).

Disinfection *in situ* has been previously reported for clayware-based MFCs, but with the use of hyperchlorite^40^ or with added antibiotics^41^. In contrast, this work has shown the *in situ* generation of a biocidal catholyte from the same energy-generating MFCs. The generated solution from wastewater without an external power supply shows a possible use of an MFC unit as an active wastewater electrolyser and possible reuse of the MFC-produced sanitising liquid as a cleaning agent or flushing system in portable urinals.

**Figure 7:**
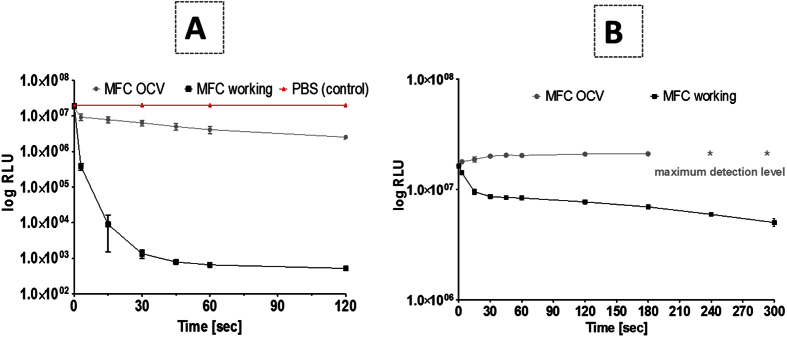
Reduction in bioluminescence from *E. coli* exposed to neat catholyte obtained from closed circuit and open circuit conditions in comparison with the control (PBS) (**A**). Reduction in bioluminescence from *E. coli* exposed to 50% catholyte at pH 7.0 obtained from closed circuit and open circuit conditions (**B**) (*shows sample overload when the measurement exceeded the measuring range of the luminometer).

Bacterial kill curves were constructed by exposing a bioluminescent *Escherichia coli* target to neat catholyte from the working MFCs (pH 11.52, conductivity 12.23 mS/cm). As can be seen in Fig. 7A, a >4-log reduction was recorded within 1 minute in comparison to the control solution (PBS), whilst a <1-log drop was recorded for the catholyte obtained from the open circuit MFCs (pH 9.7, conductivity 6.73 mS/cm).

The bioluminescence reduction could be related to the high pH of the samples, therefore, a further test was conducted. Since high pH alone is known to be biocidal, the analysis was repeated at a biocide concentration of 50% with the pH adjusted to the neutral pH level (7.0) with HCl. Figure 7B shows that the reduction in bacterial light output diminishes with time for the working MFC catholyte, while the liquid collected from the OCV MFCs shows an increase in the RLU, which eventually went above the measuring range of the luminometer. This might be due to OH– as well as H_2_O_2_ produced in the two-electron ORR pathway as previously described^42^, which if locally produced on the MFC cathode, helps eliminate biofilm which in turn is consistent with the recent report that electrochemically generated H_2_O_2_ near biofilm surfaces can efficaciously eliminate the biofilm^43^. High pH conditions also convert NH_4_^+^ to NH_3_-a chemical species known to inactivate many organisms-therefore this might also contribute to the disinfection mechanism.

These results support the hypothesis that catholyte obtained from electricity-producing MFCs has the biocidal properties and can therefore be considered as an important finding that requires further investigation.

### Ammonia based carbon capture

Ammonium bicarbonate salt solutions have shown the potential to capture salinity-gradient energy in ion-exchange membrane stacks in microbial reverse-electrodialysis cells^44^. For example, a microbial electrolysis cell with forward osmosis (MEC-FO) has been previously reported, where self-supplied ammonium bicarbonate has been shown as catholyte drag solute^45^. In the present study, as well as previously reported^29^, the conductivity of the catholyte collected and directly extracted from the anolyte, suggests that it is actively transported and related to the level of electricity produced. The increased conductivity of the newly formed catholyte suggests an increased salt concentration and it is valid to assume that this is due to the carbonate content formed as a result of carbon capture by ammonium based solutions^46^. This method may be a more cost-effective and environmentally friendly method for CO_2_ sequestration into ammonium bicarbonate, which could be used as a crop fertiliser^47^.

The quality of the liquid sample and the microbiological assessment shows that the biofilm growth in the cathode compared to the control was limited to a low concentration of specialised alkalophilic species; however the killing potency of the catholyte could be efficacious to wider populations of mesophilic bacteria, including potential pathogens. The liquid catholyte could therefore be ideal for use as flush water/disinfectant and/or cleaning agent for urinals in remote locations. This work is showing an alternative and cost effective approach with a simplified operation. Furthermore, from a future perspective, the MFC described is simple to scale up via unit replication, which can then be applied for real world applications treating real wastewater such as neat urine. The MFC design and configuration allows for immersion of the anodic compartment directly into the feedstock tank, while the open-to-air interior cathode collects the caustic effluent and performs ammonia stripping. It is envisaged that this approach will contribute to the reduction of water consumption and energy usage to recycle wastewater or urine for water, electricity and nutrients. The catholyte would be possible to use in remote waterless urinals.

### Outlook

The movement of electrons from anode to cathode drives the transport and recovery of ammonium at the cathode whilst simultaneously producing usable levels of electricity. As a result, water transport from the anode to the cathode is electroosmotically activated. Electroosmosis and subsequent electrode flooding are regarded as major problems in conventional chemical fuel cells, however for biological fuel cells coupled with water diffusion through a membrane, this could become an important benefit for water/nutrient recovery. The internal cathode design as well as activated carbon cathode have already proven their practical and cost effective advantages for real life applications^29^,^31^,^32^, while ceramic based reactors have been shown as a viable low-cost substitute to commercially available proton exchange membranes in multiple studies^48^,^49^,^50^ which makes the MFC technology accessible in developing countries^51^. The cost and sustainable operation are both extremely important from the point of view of sanitation in the Developing World as well as improvement of power generation for MFCs used in smart toilet systems. The MFC units presented in this study have also been employed in modular MFC stacks, which converted urine to electricity^31^. Utilisation of the cathodic chamber for disinfection and/or cleaning can significantly reduce the costs of urinal maintenance in remote locations, where the MFC technology may contribute towards energy efficient water recycling.

### Summary

This work describes MFCs designed to allow collection and harvesting catholyte solution to show that catholyte generation was proportional to the level of electrical current. The catholyte properties include high pH and increased conductivity in comparison to the processed wastewater and for the first time, it shows microbial growth limiting properties and supressed biofilm development on the cathode electrode. The MFC based electrolyser may be used for catholyte generation, which in turn can be reused as a biocidal and sanitising agent. This can improve the economic aspect of wastewater treatment, reclaim nitrogen as a product, remove toxic elements of ammonia from the environment and most importantly generate catholyte for ammonia recovery with net electricity gain-not loss.

## Methods

### MFC reactor construction and operation

The MFCs have been assembled using terracotta cylinders sealed at one end (Orwell Aquatics, UK) with the following dimensions: length 100 mm, outside diameter 42 mm, inside diameter 36 mm, wall thickness 3 mm as previously described^29^. The anode electrode was made of carbon veil (carbon loading 20 g/m^2^) with a macro surface area of 2430 cm^2^, which was folded and wrapped around the terracotta tube with the use of nickel chromium (Ni-Cr) wire for current collection. The cathode was made of activated carbon (30% wet proofed with PTFE) as previously described^29^. The 90 cm^2^ activated carbon cathode was inserted into the cylinder and connected via the Ni-Cr wire and stainless steel crocodile clip. The whole reactor was placed in the plastic container where the outer anode surface was fully immersed into the anolyte. Ni-Cr wire was used to connect both electrodes to the multi-channel Agilent 34972A (Farnell, UK) logging device and the electrical load. The set-up included three MFCs operated under external load (T1-T3) and three MFCs (T4-T6) used as a control group, operated under open circuit conditions. The MFC anodes had already been established over a 12-month period, as part of previous experiments, whose inoculation was with activated sewage sludge (Wessex Water Scientific Laboratory, UK). The influent consisted of a 23 mM ammonium acetate (Fisher Scientific, UK) mixed with activated sludge used as a background nutrient solution for the power evaluation experiment. The same mixture but with a concentration of 0.1 M ammonium acetate, was used for the Total Nitrogen (TN) and Chemical Oxygen Demand (COD) analysis. Ammonium acetate was chosen as a model compound to assess nitrogen recovery.

### Analysis

Total Nitrogen was measured using MD500 colorimeter (Lovibond, UK) and Vario Tube Test (0.5–25 mg/L) using diluted samples. For measuring chemical oxygen demand (COD), samples were filtered through 0.45 µm filters (Millex, USA) and then analysed using MD 200 photometer (Lovibond, UK) and potassium dichromate oxidation test vials (COD HR, Camlab, UK). A Hanna 8424 pH meter (Hanna, UK) was used for the pH measurements and a Jenway conductivity meter (Camlab, UK) with an operating range of 0–1999 mS/cm was used for conductivity measurements.

The total viable count (TVC) of organisms in catholyte samples was performed by a conventional serial dilution method with surface spreading of 0.1 mL dilution samples onto nutrient agar (peptone, yeast extract and salt based agar, Oxoid, UK) petri dishes, which were incubated aerobically at room temperature (22 °C) for 48 h. Microbial colony counts are expressed as log_10_ colony forming unit (CFU) per sample of catholyte.

### Single tube luminometric assay

The catholyte was freshly collected from the reactors and tested as a biocide. Bioluminescent *Escherichia coli* Nissle 1917 pGLITE was grown on nutrient agar plates supplemented with kanamycin (10 μg/mL) to maintain the lux plasmid and further transferred to Brain-Heart infusion liquid broth for 3 hours incubation at 37 °C to enable culture growth to mid-exponential phase prior to use in the experiment. Subsequently, 0.1 mL of bacterial culture was added to a clean borosilicate glass tube (12 by 75 mm; Fisher Scientific, Loughborough, United Kingdom) for bioluminescence target organisms measurement using a single-tube FB12 luminometer (Berthold Detection Systems, Germany) to quantify relative light units (RLU) in 1 mL of phosphate buffer saline (PBS pH 7.4) as the control and to the same amount of catholyte (diluted where appropriate) to evaluate the biocidal efficacy. The automated protocol included a 3s delay to allow the reading of measurements. Bacterial bioluminescence was recorded every 15s for the first minute and then at 120s intervals. Units of relative light emission (RLU) were transformed to log_10_ values and plotted to show kill kinetics of the target organism. Light output from bioluminescent bacteria can be used to monitor real-time effects of antimicrobial agents due to the strong correlation between bioluminescence and metabolic activity, which infers culture viability^52^.

## Additional Information

**How to cite this article**: Gajda, I. *et al*. Electricity and disinfectant production from wastewater: Microbial Fuel Cell as a self-powered electrolyser. *Sci. Rep.* **6**, 25571; doi: 10.1038/srep25571 (2016).

## Supplementary Material

Supplementary Information
