# Supplementary material for: Electricity and disinfectant production from wastewater: Microbial Fuel Cell as a self-powered electrolyser
Source: Sci Rep. 2016 May 12;6:25571. doi: 10.1038/srep25571 (PMC4865956; doi:10.1038/srep25571)
Supplement: Supplementary Information [file srep25571-s1.docx]

**Supplementary Information**

**Electricity and disinfectant production from wastewater**: **Microbial Fuel Cell as a self-powered electrolyser**

Iwona Gajda^a,*^, John Greenman^a,b^, Chris Melhuish^a^, Ioannis A. Ieropoulos^a,b*^

^a^ Bristol BioEnergy Centre, Bristol Robotics Laboratory, University of the West of England, BS16 1QY, UK

^b^ Biological, Biomedical and Analytical Sciences, University of the West of England, BS16 1QY, UK

^*^Corresponding authors: [Iwona.Gajda@uwe.ac.uk](mailto:Iwona.Gajda@uwe.ac.uk), [Ioannis.Ieropoulos@brl.ac.uk](mailto:Ioannis.Ieropoulos@brl.ac.uk)


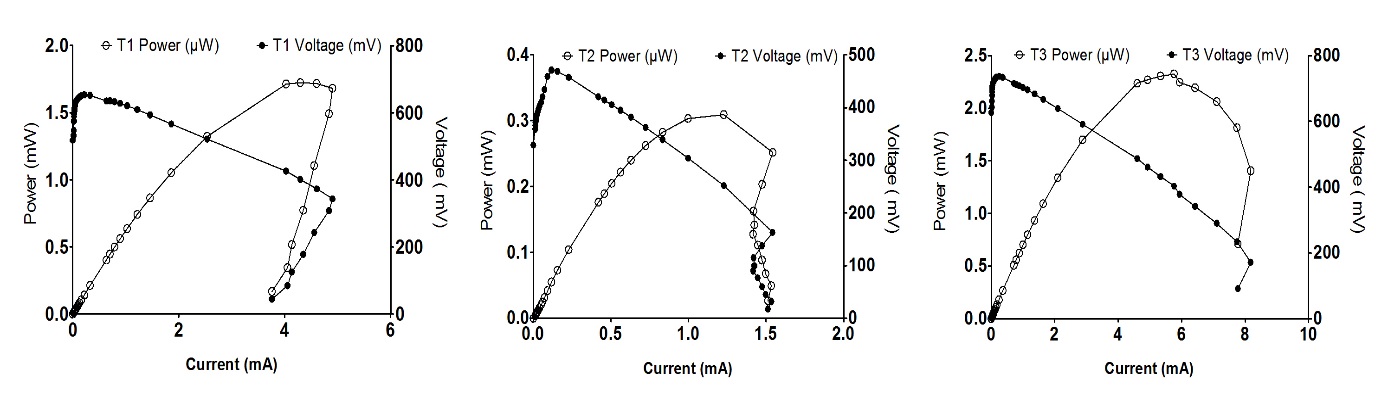


Figure S1. Polarisation data performed on MFCs: T1, T2 and T3 applying a resistance value every 3-minute intervals.
